# Molecular spectrum of TSHβ subunit gene defects in central hypothyroidism in the UK and Ireland

**DOI:** 10.1111/cen.13149

**Published:** 2016-08-04

**Authors:** A.K. Nicholas, S. Jaleel, G. Lyons, E. Schoenmakers, M.T. Dattani, E. Crowne, B. Bernhard, J. Kirk, E.F. Roche, V.K. Chatterjee, N. Schoenmakers

**Affiliations:** ^1^ University of Cambridge Metabolic Research LaboratoriesWellcome Trust‐Medical Research Council Institute of Metabolic ScienceAddenbrooke's HospitalCambridgeUK; ^2^ Department of Paediatric Endocrinology & DiabetesNational Children's HospitalAMNCHDublinIreland; ^3^ University College London Institute of Child HealthDevelopmental Endocrinology Research GroupSection of Genetics and Epigenetics in Health and DiseaseGenetics and Genomic Medicine ProgrammeLondonUK; ^4^ Department of Paediatric Endocrinology & DiabetesBristol Royal Hospital for ChildrenUniversity Hospitals Bristol NHS Foundation TrustBristolUK; ^5^ Department of Clinical GeneticsNorth West Thames Regional Genetics ServiceNorth West London Hospitals NHS TrustHarrowUK; ^6^ Department of EndocrinologyBirmingham Children's HospitalBirminghamUK; ^7^ University of DublinTrinity College DublinDublinIreland

## Abstract

**Objective:**

Homozygous mutations in the TSH beta subunit gene (*TSHB*) result in severe, isolated, central congenital hypothyroidism (CCH). This entity evades diagnosis in TSH‐based congenital hypothyroidism (CH) screening programmes in the UK and Ireland. Accordingly, genetic diagnosis, enabling ascertainment of affected relatives in families, is critical for prompt diagnosis and treatment of the disorder.

**Design, Patients and Measurements:**

Four cases of isolated TSH deficiency from three unrelated families in the UK and Ireland were investigated for mutations or deletions in *TSHB*. Haplotype analysis, to investigate a founder effect, was undertaken in cases with identical mutations (c.373delT).

**Results:**

Two siblings in kindred 1 were homozygous for a previously described *TSHB* mutation (c.373delT). In kindreds 2 and 3, the affected individuals were compound heterozygous for *TSHB* c.373delT and either a 5·4‐kB *TSHB* deletion (kindred 2, c.1‐4389_417*195delinsCTCA) or a novel TSHB missense mutation (kindred 3, c.2T>C, p.Met1?). Neurodevelopmental retardation, following delayed diagnosis and treatment, was present in 3 cases. In contrast, the younger sibling in kindred 1 developed normally following genetic diagnosis and treatment from birth.

**Conclusions:**

This study, including the identification of a second, novel, *TSHB* deletion, expands the molecular spectrum of *TSHB* defects and suggests that allele loss may be a commoner basis for TSH deficiency than previously suspected. Delayed diagnosis and treatment of profound central hypothyroidism in such cases result in neurodevelopmental retardation. Inclusion of thyroxine (T4) plus thyroxine‐binding globulin (TBG), or free thyroxine (FT4) in CH screening, together with genetic case ascertainment enabling earlier therapeutic intervention, could prevent such adverse sequelae.

## Introduction

Isolated congenital central hypothyroidism is rare (incidence 1 in 65 000),^1^ with genetic causes comprising mutations in the TSH beta subunit (*TSHB*), the immunoglobulin superfamily member 1 (*IGSF1*) or, less frequently, the TRH receptor (*TRHR*) genes.^2^ Central hypothyroidism is associated with either subnormal or inappropriately normal TSH levels and therefore evades diagnosis in the UK and Irish TSH‐based newborn screening programmes for congenital hypothyroidism (CH). Unfortunately, patients may then present later in the neonatal period with overt clinical hypothyroidism; in *TSHB* mutation cases, thyroid hormone deficiency is profound, resulting in neurodevelopmental retardation.^3^

Heterodimeric TSH, secreted by anterior pituitary thyrotroph cells, comprises a hormone‐specific beta subunit (TSHβ) and a common alpha subunit (αGSU) shared with other members of the glycoprotein hormone family (luteinizing hormone, follicle‐stimulating hormone and chorionic gonadotrophin). The integrity and bioactivity of the heterodimer are maintained by a ‘seat belt’ structure formed by the β‐subunit peptide that is stabilized by disulphide bridges including the so‐called buckle between amino acids 39 and 125.^4^

The human TSHβ subunit gene is organized into 3 exons: exon 1 is untranslated, and exons 2 and 3 encode a 138‐amino acid peptide. The twenty N‐terminal amino acids encode a signal peptide that is cleaved to yield a 118‐amino acid mature protein detectable in serum.^4^, ^5^ The most frequently described *TSHB* mutation is a single‐nucleotide deletion (c373delT), resulting in a cysteine 125 to valine conversion (C125V) and disruption of the Cys‐Cys 39–125 disulphide bridge, with a subsequent shift in reading frame and premature stop codon at position 134.^6^ Eight additional mutations have been described, including missense (p.C108Y, p.C105R, p.G49R) and nonsense or frameshift mutations (p.E32*, p.Q69*, p.F77Sfs*6).^3^, ^7^, ^8^, ^9^, ^10^, ^11^ Two splice site mutations (c162G>A, IVS2 + 5 G>A),^3^, ^12^ and more recently a homozygous *TSHB* deletion, have also been reported.^13^ All are inherited in an autosomal recessive manner and are associated with severe central hypothyroidism. (The nomenclature of mutations in this paper follows the most recent HGNC guidelines to include the 20‐amino acid signal peptide of TSHβ, such that codon numbering may differ from that cited in the previously published articles).

We report three kindreds from the UK or Ireland in which four cases exhibit isolated central hypothyroidism secondary to *TSHB* mutations. In kindred 1, two siblings (P1a and P1b) are homozygous for the previously described c.373delT mutation. In kindred 2, the affected child (P2) is compound heterozygous for c.373delT together with a maternally derived 5·4‐kb deletion involving *TSHB* alone (c.1‐4389_417*195delinsCTCA). In kindred 3, the proband (P3) is compound heterozygous for c.373delT and a novel missense mutation (c.2T>C, p.Met1?), disrupting the methionine residue from which translation is initiated. We describe these *TSHB* defects and associated clinical phenotypes in the context of the wider literature.

## Patients and methods

Clinical phenotype, auxological parameters and biochemistry for each case are summarized in Table 1.

**Table 1:** Clinical phenotype, auxological and biochemical parameters for each case harbouring *TSHB* mutations

	P1a	P1b	P2	P3
Neonatal screening TSH (mU/l)	‘Normal’	‘Normal’	<1	0
Gestational length (weeks)	42	42	40	39
Birth weight (kg)	3·71	4·14	3·18	2·8
At diagnosis				
Age (weeks)	5	Birth	8	14
Body length (cm) and centile	56·2 (25th)	NA	51 (<3rd)	<3rd
Body weight (kg) and centile	5·60 (75th)	4·14 (75th)	3·52 (<3rd)	NA(3rd)
TSH (mU/l)	0·1(0·1–5·0)	<0·05(0·3–4·0)	2·78 (0·4–4·0)	0·45 (0·4–3·5)
fT4 (pmol/l)	<10(50–160)^a^	6·5(10–24)	<3·89(10–25)	<5·1 (13·8–22·5)
Peak TSH response to TRH	0·09	NA	3·21	NA
MRI pituitary	Normal	NA	Normal	Normal
Extrathyroidal abnormalities				
Neurodevelopment	Mild motor and sensory processing deficits, extra support at school	Normal	Mild motor delay	Horizontal pendular nystagmus, bilateral sensorineural hearing loss developmental delay (motor, language), Autism/Asperger spectrum Attends special school
Haematological			Macrocytic anaemia	Macrocytic anaemia

a In P1a, the T4 value refers to total T4 (nmol/l). In three cases, the absolute T4 value could not be quantified because it was significantly below the lower limit of the normal reference range (P1a, 50–160 nmol/l, Ciba Corning ACS 180) or reportable range quoted by the assay manufacturer (P2 3·9pmol/l, Siemens Immulite 2500 SMS, P3 5·1pmol/l, Abbott AxSym Immunoassay).

### Patients 1a and 1b (P1a and P1b)

P1a is the second son of healthy, nonconsanguineous Caucasian (UK) parents. Although his TSH on neonatal screening was reported normal, he presented aged 3 weeks with a lower respiratory tract infection and was noted to have clinical features of hypothyroidism, including somnolence, constipation and dry skin. Thyroid function tests were consistent with severe central hypothyroidism (total T4 < 10 nmol/l, normal range 50–160; TSH 0·1 mU/l, NR 0·1–5), with no TSH response to TRH stimulation. Subsequent dynamic testing, showing preserved corticotroph, somatotroph and gonadotroph function, excluded combined pituitary hormone deficiency (data not shown), and his basal prolactin level was 554 mU/l (NR <700). Levothyroxine treatment was commenced at the age of 5 weeks and 2 days, despite good compliance development was delayed. Formal assessment of age 2 years revealed significantly impaired hearing and speech. At age 7 years, growth is normal, and he is in mainstream education, but requires additional support.

P1b, the younger brother of P1a, underwent screening for central hypothyroidism at birth following this diagnosis in his older sibling. Thyroid function was consistent with TSH deficiency (cord blood fT4 6·5 pmol/l, NR 10–24; TSH <0·05 mU/l, NR 0·3–4). Levothyroxine was commenced at age 1 week, and he subsequently grew and developed normally. Both parents are clinically and biochemically euthyroid.

### Patient 2 (P2)

P2 is the daughter of healthy, nonconsanguineous Caucasian (Irish) parents. Her TSH in neonatal screening was normal. She presented aged 8 weeks with prolonged jaundice, poor weight gain and feeding since birth, somnolence and constipation. She was clinically hypothyroid, with cool peripheries, hypotonia, an umbilical hernia, cutis marmorata, coarse facial features and macroglossia. Severe biochemical central hypothyroidism was confirmed (fT4 < 3·89 pmol/l, NR 10–25; TSH 2·78 mU/l, NR 0·4–4·0). In a TRH stimulation test, the TSH response was absent, but prolactin levels rose (basal prolactin 723 mU/l, 20 min prolactin 1064 mU/l), suggesting a defect downstream of the TRH receptor. Dynamic evaluation of corticotroph and somatotroph function was normal. Following commencement of levothyroxine aged 8 weeks, her growth was normal. Despite good compliance with treatment, she exhibits mild motor delay at age 7 years. Both parents are clinically and biochemically euthyroid.

### Patient 3 (P3)

P3 is the only daughter of healthy, nonconsanguineous Caucasian (UK) parents. Her TSH in neonatal screening was reported as normal. She presented aged 3·5 months with feeding difficulties, somnolence, constipation and severe growth retardation. She was clinically hypothyroid with a small umbilical hernia and depressed nasal bridge. Thyroid biochemistry confirmed profound central hypothyroidism (fT4 < 5·1 pmol/l, NR 13·8–22·5; TSH 0·45 mU/l, NR 0·4–3·5). Subsequent evaluation of pituitary–adrenal, gonadotrophins and basal serum prolactin was normal. Although her growth response to levothyroxine treatment was excellent, she has multiple neurodevelopmental deficits including bilateral sensorineural deafness and nystagmus without optic nerve hypoplasia. Motor and language development was significantly delayed; at the age of 10 years, she is on the autistic/Asperger's spectrum. She has a statement of Special Educational Needs and is unable to attend mainstream school. Her mother is clinically and biochemically euthyroid; her father is not available for evaluation.

## Methods

All investigations were part of an ethically approved protocol and/or clinically indicated, being undertaken with the consent from patients and/or next of kin.

### Sanger sequencing of the *TSHB* subunit gene

Genomic DNA was extracted from peripheral blood leucocytes using the standard techniques. All three *TSHB* subunit gene exons and exon/intron boundaries were amplified by PCR using specific primers: 5’‐TCCAGGTAAAGATATTGTGAGCT‐3’ and 5’‐TTACAGCCTGTTGAAGCAAATT‐3’ (exon 1), 5’‐CCTAGATTTCTGAGTTAGCCCCT‐3’ and 5’‐TGCGTATCCATTGTGCTGAG‐3’ (exon 2) and 5’‐TGTTTCCTAAAGTCCTGTCACA‐3’ and 5’‐ACCTTCAACTGAGCCCAAAAG‐3’ (exon 3). To characterize the chromosomal deletion around *TSHB* in P3, the homozygous region delineated by SNP markers rs7523360 and rs1998008 was amplified by long‐range PCR using the primers 5’‐AACACACATCCAACATTCTTCCA‐3’ and 5’‐AAATCCCGCAGTAATTCTTTGCT‐3’.

PCR products were sequenced using the BigDye Terminator v3.1 Cycle Sequencing Kit (Applied Biosystems, Foster City, USA) and 3730 DNA Analyzer (Applied Biosystems). The *TSHB* variants listed in this study are described using the systematic nomenclature approved by the Human Genome Variation Society (HGVS; varnomen.hgvs.org). Nucleotide numbering starts from the A (+1) of the translation initiation codon (ATG) of the NCBI reference sequence NM_000549.4 and includes the 20‐amino acid signal peptide. Amino acid residues are numbered according to the NCBI reference sequence NP_000540.2.

### Haplotype analysis

To determine chromosome 1 haplotypes, individuals were genotyped for single‐nucleotide polymorphisms with MAF >0·38 from seven linkage disequilibrium blocks selected using Haploview (The Broad Institute, USA) and spanning 225 kb 5’ and 205 kb 3’ of the *TSHB* locus (Figure 3, primers available on request).

### *In silico* tools

*In silico* analysis of the novel missense variant (c.2T>C) was performed using Alamut as an interface (Interactive Biosoftware, Rouen, France) and the SignalP V4.1 signal peptide prediction package (www.cbs.dtu.dk/services/SignalP) to determine whether translation from Met‐7 would alter the probability of the protein sequence acting as a signal peptide.^14^

### TaqMan quantitative real‐time PCR (qPCR) analysis of *TSHB* gene copy number

The following intra‐exonic primer/probe sets were used to quantify genomic *TSHB* copy number and genomic *THRA* in P3, her parents, and 10 unrelated, euthyroid controls. *TSHB*: primer/probe set Hs02759015_s1 (ABI Life Technologies); THRA: forward 5’‐TTCGAGCACTACGTCAACCA‐3’; reverse 5’‐CCCGATCATGCGGAGGTCAG‐3’; probe 5’‐FAM CACAACATTCCGCACTTCTG‐TAMRA‐3’ using qPCR as previously described.^15^ The comparative Ct method was used to quantify genomic *TSHB* normalized to genomic *THRA*.

## Results

### *TSHB* analysis using Sanger sequencing

**Figure 1:**
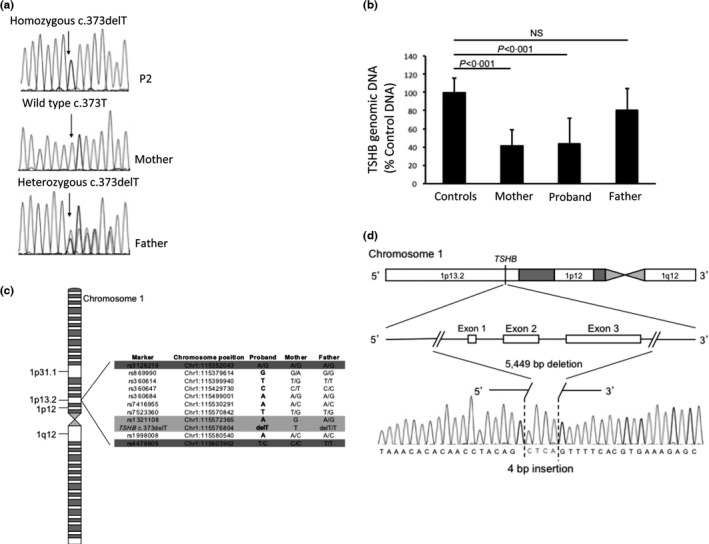
(a) Apparent uniparental inheritance of the *TSHB* c.373delT mutation in P2: Chromatograms obtained from *TSHB* sequencing in P2 and parents; P2 is homozygous for a single‐nucleotide deletion at position 373 (c.373delT), and her father is heterozygous for the mutation as predicted. Surprisingly, the maternal sequence is apparently wild type. (b) Decreased genomic *TSHB* in P2 and her mother are consistent with a maternally inherited *TSHB* deletion: TaqMan qPCR was used to quantify genomic *TSHB* normalized to genomic *THRA* levels and expressed as a percentage of the mean for 10 euthyroid controls; levels in P2 and her mother are approximately fifty per cent of those in her father and controls, consistent with a heterozygous maternal genomic deletion at the *TSHB* locus. *P*‐values were computed using a 2‐tailed Student's *t*‐test. (c) Single‐nucleotide polymorphism (SNP) genotyping around the *TSHB* locus defines the deleted region in P2: Schematic summarizing SNP genotyping around the *TSHB* locus. Light grey: SNPs with obligate uniparental inheritance; dark grey: heterozygous SNPs defining the boundaries of obligate diparental inheritance. Single bases in P2 define the region of loss of heterozygosity. (d) Maternally inherited deletion–insertion in P2: Schematic of the *TSHB* locus and sequencing chromatogram defining the maternal deletion–insertion for which P2 was compound heterozygous.

P1a and P1b were homozygous for a single‐nucleotide deletion in *TSHB*, c.373delT, p.C125Vfs*10 (c.313delT, p.C105Vfs114X, legacy numbering), described previously, with both euthyroid parents being heterozygous. Despite paternal heterozygosity for this mutation, but wild‐type maternal *TSHB* sequence (Fig. 1a), P2 was apparently homozygous for the same mutation (p.C125Vfs*10). This suggested uniparental inheritance of the mutation, due to either paternal, uniparental disomy or a maternal deletion involving the *TSHB* locus. P3 was heterozygous for the p.C125Vfs*10 mutation and a novel missense mutation (c.2T>C, p.Met1?), disrupting the start site for translation at the beginning of the signal peptide of TSHβ. As the maternal *TSHB* locus only showed heterozygosity for the c.2T>C, p.Met1? change, these mutations were assumed to be compound heterozygous, occurring on separate alleles.

### Investigation to define the *TSHB* deletion in P2

TaqMan qPCR was used to quantify *TSHB* copy number in P2, her parents, and ten unrelated, euthyroid controls. The results, showing a fifty per cent decrease in amplified signal from the *TSHB* locus in P2 and her mother, were consistent with a maternal, heterozygous *TSHB* deletion (Fig. 1b).

P2 and both parents were genotyped for single‐nucleotide polymorphisms around the *TSHB* locus. This analysis delineated a 211‐Kb region with loss of heterozygosity at 1p13·2, with heterozygous SNPs confirming obligate biparental inheritance at the boundaries of this interval. An informative SNP (rs1321108), 4·4 kb proximal to *TSHB*, confirmed uniparental (paternal) inheritance at this site (A/A), as no maternal allele (G/G) was inherited (Fig. 1c). Subsequent Sanger sequencing of the region, using a long‐range PCR, confirmed a 5·4‐kb maternal deletion involving only the *TSHB* locus, together with a 4‐bp insertion (c.1‐4389_417*195delinsCTCA) (Fig. 1d).

### *In silico* prediction of the deleterious consequences of the c.2T>C mutation in P3

P3 exhibits a novel missense mutation (c.2T>C), in which a methionine codon, from which translation is initiated, is replaced by a threonine. This mutation was absent from the published normal genome data sets (dBSNP, 1000 Genomes, Exome Aggregation Consortium (ExAC), Cambridge, MA (URL: http://exac.broadinstitute.org), April 8th, 2016). Alamut predictive software suggests that this substitution will adversely affect translation of the TSHβ polypeptide.

**Figure 2:**
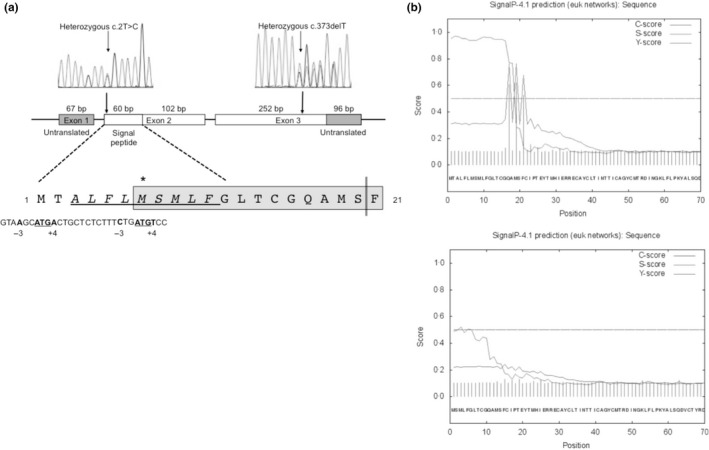
(a) Schematic summarizing the deleterious c. 2T>C and c.373delT mutations in P3: Sequencing chromatograms are aligned with a schematic of the *TSHB* gene to show the position of the compound heterozygous mutations. The amino acid sequence of the TSHB signal peptide contains a putative hydrophobic core (underlined) and cleavage site (double vertical line). Translation from Met‐7 due to the c.2T>C mutation would result in loss of the N‐terminal portion of the peptide and hydrophobic residues, with the resultant amino acid sequence, highlighted in grey. The cDNA nucleotide sequence around Met‐1 and Met‐7 is shown beneath, with the bases at positions ‐3 and +4 in bold type, defining favourable (Met‐1) and unfavourable (Met‐7) Kozak sequences for translation initiation. (b) Translation from Met‐7 is likely to abrogate the *TSHB* signal peptide: Signal P 4.0 analysis output for wild‐type *TSHB*, and *TSHB* lacking the first 6 amino acids (i.e. the putative peptide generated if translation occurs from Met‐7). The presence of a signal peptide is predicted by scoring every residue (‘S‐score’) for the stretch of amino acids analysed. The predicted score for the signal peptidase cleavage site is indicated by the C‐score. Y‐score is a composite score for C‐ and S‐scores, to improve cleavage site prediction. The D‐score (discrimination score) is a weighted average of the mean S and the max. Y scores and is used to discriminate signal peptides from nonsignal peptides, with a value of >0·45 taken to predict a signal peptide (www.cbs.dtu.dk/services/SignalP-4.0/output.php). Wild‐type TSHB (upper panel) is strongly predicted to contain a cleavable signal peptide within residues 1–20 (C‐score 0·62, Y‐score 0·76, S‐score 0·97, D‐score 0·853), whereas this prediction becomes much weaker when Met‐7 is used as the translational start site (lower panel), and no clear cleavage site is denoted (C‐score 0·13, Y‐score 0·24, S‐score 0·52, D‐score 0·334).

In higher eukaryotes, efficient initiation of translation is governed by the nucleotide sequence surrounding the ATG start codon (GCCGCC**A/G**CC*A*UG **G,** initiator codon underlined), with a purine (usually A) at position ‐3 (5’) and guanine at position +4 (3’), relative to the adenine (A) of the initiator codon (Fig. 2a).^16^ Thus, although there is an alternative, in‐frame, ATG start codon at Met‐7, the nucleotide sequence surrounding Met‐7 (CTCTTT**C**TG*A*TG **T**) lacks both a purine at position ‐3 (C, bold) and a guanine at position +4 (T, bold), suggesting that efficient translation from this alternative site is unlikely. Additionally, as the first 20 amino acids of TSHβ form a signal peptide, even if limited synthesis from Met‐7 of TSHβ occurs, the resulting N‐terminally deleted TSHβ polypeptide would lack hydrophobic residues within its signal sequence that may be critical for correct protein translocation (Fig 2a).^17^ SignalP 4.1 software^14^ also predicts that if translation occurred from Met‐7, the ability to appropriately cleave signal peptide from TSHβ polypeptide would be lost (Fig. 2b). Taken together, these features suggest that the c.2T>C *TSHB* mutation is likely to result in significantly impaired biosynthesis of functional TSHβ polypeptide.

### Haplotype analysis around the *TSHB* c.373delT mutation

**Figure 3:**
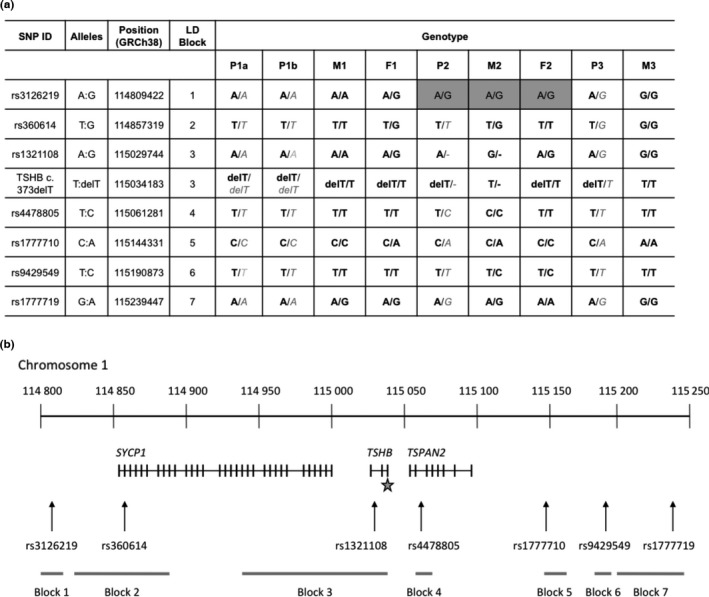
(a) The c.373delT mutation occurs on a common haplotype in all 3 kindreds: Table showing the chromosomal location of genotyped SNPs, and the genotype of probands and parents. The maternal alleles inherited by the probands are shown in grey italics. P: proband; M: mother; F: father; cells with grey fill: inheritance unclear. (b) Schematic showing the chromosomal location of genotyped SNPs, within seven blocks 1–7 that are in tight linkage disequilibrium and their position relative to *TSHB* and neighbouring *SYCP1* and *TSPAN2* genes. The location of the c.373delT mutation is denoted by a star.

Genotyping for single‐nucleotide polymorphisms around *TSHB* indicates that all four cases share an extended haplotype for the c.373delT mutation, suggesting common ancestry (Fig. 3a,b). Intriguingly, all of the UK cases may be of Irish descent; P3, with presumed paternal inheritance of c.373delT, confirms paternal Irish descent; P1a and P1b also acknowledge paternal Irish ancestry.

## Discussion

This report represents first documentation of three different *TSHB* mutations in patients from the UK and Ireland exhibiting characteristic clinical features, although two American cases of Scottish–Irish ancestry have been described.^18^ Neurodevelopmental retardation is a consistent feature in *TSHB* mutation cases, often attributed to delayed diagnosis and initiation of treatment, as these individuals are not detected in TSH‐based, CH screening programmes.^3^ Thus, in our patients, severity of neurodevelopmental impairment correlates with the age of thyroxine commencement: in P3, who was diagnosed aged 3·5 months, neurodevelopmental retardation is most marked, and she requires special schooling; despite initiation of treatment aged 6 or 8 weeks, respectively, P1a and P2 both exhibit mild neurodevelopmental impairment; in P1b, who was screened, diagnosed and treated at birth, neurological development was normal.

P3 exhibits a constellation of neurological deficits, much of which can be explained by early postnatal hypothyroxinaemia. Sensorineural hearing loss has been described in association with both primary and secondary hypothyroidism 3, 19, 20 and reflects the essential role of thyroid hormone in the auditory development for maturation and innervation of cochlear hair cells, and development of central auditory pathways.^21^, ^22^ Additionally, nystagmus has been reported in the context of primary congenital hypothyroidism 23, 24 and, given the importance of thyroid hormone in postnatal cerebellar granule cell development, may indicate defective maturation of cortico‐pontine‐cerebellar pathways.^25^ The complex neurodevelopmental abnormalities in P3 include autistic spectrum disorder, which, being reportedly associated with hypothyroxinaemia *in utero* rather than postnatally,^26^ might not be aetiologically linked to her congenital TSH deficiency, although a role for early postnatal hypothyroxinaemia may be plausible. Given the crucial postnatal role of thyroxine in the development of visuospatial, language and sensorimotor skills, central hypothyroidism is likely to have contributed to her neurodevelopmental delay.^25^

Our cases exhibit three different genetic aetiologies including one novel missense *TSHB* mutation (P3, c.2T>C, p.Met1?). Mutation of a normal mammalian ATG start codon to ACG usually results in markedly diminished protein translation 27 and thus would be expected to cause significantly reduced synthesis of full‐length TSHβ polypeptide from the mutant allele. Another in‐frame ATG codon, six residues downstream (Met‐7), is surrounded by an unfavourable nucleotide context for translational initiation, lacking both a purine at position ‐3 and a guanine at position +4.^16^ Even if limited TSHβ synthesis from Met‐7 occurred, this polypeptide would likely lack a recognizable signal sequence, compromising TSHβ processing and secretion.^17^ Consistent with such *in silico* predictions, *in vivo* data from P3 strongly suggest that negligible bioactive TSH is synthesized from the c.2T>C allele, as profound hypothyroidism (undetectable fT4) is associated with barely measurable serum levels (0·45 mU/l) of intact TSH. Some of this TSH immunoreactivity may reflect translation of TSHβ from the residual c.373delT *TSHB* mutant allele, which is known to mediate the limited synthesis of biologically inactive TSH that retains immunoreactivity in some assays.^19^

P2 is the first recorded patient in whom a heterozygous deletion (5·4 kb, c.1‐4389_417*195delinsCTCA), encompassing *TSHB* alone, has been mapped precisely. Biallelic *TSHB* deletions were recently described in a Turkish patient.^13^ Intriguingly, one of the original Brazilian cases with a c.373delT *TSHB* mutation exhibited apparent uniparental inheritance; compound heterozygosity of the c.373delT mutation with a *TSHB* deletion would also be a plausible alternative explanation in this patient.^6^ P2 harbours a heterozygous *TSHB* deletion together with a monoallelic c.373delT mutation, suggesting that *TSHB* deletions may be a more common cause of isolated TSH deficiency than previously thought.

Worldwide, c.373delT is the commonest *TSHB* mutation reported, occurring in the unrelated cases from Western Europe, South America and the USA.^28^, ^29^, ^30^, ^31^ Accordingly, it is unsurprising that all of our cases harbour c.373delT *TSHB* either mono‐ or biallelically. Previous functional studies have shown that it is the cysteine to valine amino acid change at codon125, rather than subsequent deletion of 13 carboxyterminal residues of TSHβ, which impairs the function of mutant TSH.^6^ This amino acid substitution disrupts a disulphide bond between C125 and C39 that forms the ‘buckle’ of the TSHβ ‘seat belt’ which surrounds the αlpha subunit. Although the c.373delT mutation disrupts the functional integrity of TSH, alpha and mutant TSHβ subunits can still heterodimerize, forming biologically inactive TSH that retains immunoreactivity in some immunoassays.^19^ Consistent with this observation, the IMMULITE 2000 third‐generation immunoassay (Siemens) detects TSH in P2, albeit at inappropriately low levels. Although the c.373delT nucleotide change occurs at a mutation hot spot in *TSHB*, the gene defect is present as a founder mutation in some populations.^32^ Thus, analysis of our UK and Irish cases showed that the mutation also occured on a common (probably Irish) haplotype background in these families, supporting the notion of a founder effect.

Screening for congenital central hypothyroidism (CCH) can be achieved by inclusion of thyroxine (T4) plus thyroxine‐binding globulin (TBG), in the CH screening programme, as in the Netherlands or fT4 as in Japan. Combined T4/TSH/TBG evaluation in Dutch neonates enables diagnosis of permanent central CH with an incidence of 1:21 000 newborns.^33^ The incidence in Japan has been found to be much lower (1:160 000); however, although ethnic differences could be contributing, subsequent studies suggest this may reflect a less sensitive Japanese screening approach.^34^, ^35^

The benefits of including T4 measurement in CH screening continue to be debated with arguments against citing the relative rarity of congenital central hypothyroidism, with the presumption that it is usually mild and unlikely to be associated with adverse neurological sequelae. However, screening for CCH would enable prompt diagnosis and treatment of affected cases, improving neurodevelopmental outcomes particularly in severe cases due to *TSHB* mutations. Moreover, Zwaveling‐Soonawala *et al*. recently demonstrated that more than 50% of children diagnosed with CCH in the Netherlands exhibit moderate or severe hypothyroidism, rather than mild thyroid hormone deficits, thus emphasizing that the potential neurological sequelae of delayed diagnosis should not be underestimated (Zwaveling‐Soonawala *et al*. 2015).^36^ As combined pituitary hormone deficiencies occur in 75 per cent of CCH cases, early diagnosis of central hypothyroidism would also facilitate the prompt detection of pituitary–adrenal and growth hormone deficiencies, potentially preventing their life‐threatening consequences.^2^

We report the first cases from the UK and Ireland harbouring *TSHB* mutations, whose genetic aetiologies include a novel missense mutation, a compound heterozygous *TSHB* deletion and a known frameshift mutation. As well as expanding the known repertoire of *TSHB* mutations, our observations suggest that *TSHB* deletions may be more common than previously thought and show that c.373delT *TSHB* mutation is the most common genetic defect in cases of central hypothyroidism in the UK and Ireland. Concordant with other recorded cases, our patients with isolated TSH deficiency exhibit neurodevelopmental delay when overt hypothyroidism is diagnosed and treated later postnatally. As measurement of T4 is not included in UK or Ireland CH screening programmes, genetic ascertainment in families with isolated TSH deficiency will be particularly important, enabling prompt diagnosis of affected children and early treatment to prevent the adverse neurodevelopmental outcome.

## Disclosure

Nothing to declare.
